# The Effect of Elderly Patients’ Health Information Literacy, Ageism, and Communication Skills on Clinical Nurses’ Burnout: A Cross-Sectional Study

**DOI:** 10.3390/nursrep16020045

**Published:** 2026-01-29

**Authors:** Eunhee Shin

**Affiliations:** Department of Nursing Science, Sangji University College of Health Sciences, Wonju 26339, Republic of Korea; hshin1970@sangji.ac.kr; Tel.: +82-33-738-7623

**Keywords:** health literacy, communication skills, ageism, burnout, clinical nurses

## Abstract

**Background:** This study aimed to examine correlation between nurses’ assessments of health literacy in older adults, communication skills, and ageism, as well as whether these factors could be key predictors of nurse burnout. **Methods:** To determine which factors predict burnout among clinical nurses, a structured questionnaire was distributed to 269 clinical nurses. Data were analyzed using descriptive statistics, *t*-test, ANOVA, Pearson’s correlation coefficients, and multiple regression analysis. **Results:** Elderly patients’ health literacy assessed by nurses showed significant correlations with communication skills, ageism, and burnout. Communication skills were negatively correlated with ageism and burnout, whereas ageism showed a strong positive correlation with burnout. Multiple regression analysis revealed that ageism (β = 0.287), communication skills (β = −0.251), female gender (β = 0.139), and aging anxiety (β = −0.181)were significant predictors of burnout, collectively explaining 29.3% of the variance in burnout. **Conclusions:** Ageism was the strongest predictor of burnout among clinical nurses, followed by communication skills. Strategies reducing ageism and enhancing communication competencies are essential for mitigating burnout in geriatric nursing practice. These findings highlight the need for systematic educational interventions related to the elderly tailored for both nursing students and clinical nurses.

## 1. Introduction

The elderly population is increasing worldwide, and approximately 73% of those aged 65 and older are reported to suffer from at least one chronic disease, such as depression or dementia [1]. Accordingly, the issues of health management and nursing for the elderly are emerging as important social issues [2]. The proportion of elderly patients visiting medical institutions is growing, and the demand for multi-dimensional health management for them is gradually increasing, resulting in a growing demand for services from medical professionals [1]. Among these, nurses are the medical professionals who have the most contact with elderly patients in clinical settings. They need to provide nursing services tailored to elderly patients in terms of physical, emotional, and social aspects. They are also a key workforce that must engage with the well-being and quality of life of the continuously increasing elderly generation and show initiative in solving their health-related problems [3].

The World Health Organization (WHO) identifies health literacy as a key determinant of health, defining it as “the personal, cognitive, and social skills that guide and determine an individual’s ability to understand and use information to promote and maintain health” [4]. Poor health literacy is known to lead to inappropriate use of medical services and inadequate disease management, negatively impacting physical and mental health and quality of life [5]. In particular, for older adults, who often experience negative health changes due to chronic diseases, disabilities, and various functional declines, acquiring and utilizing various health-related information is crucial for managing their health [6]. However, previous studies have reported that a significant number of older adults struggle to understand and utilize health information [7]. Several recent studies have reported that health literacy in older adults is significantly associated with demographic factors such as age, gender, education level, and socioeconomic status [8,9]. Higher education levels and younger age are associated with better health literacy. Therefore, elderly individuals aged 65 years or older with low education levels have very low health information literacy [8,10], and thus are considered vulnerable to misunderstanding medical information, so nurses must be mindful in this regard. Compared with patients with adequate health information literacy, those with poor health information literacy are more likely to misunderstand medical information, especially explanations related to treatment processes and medical conditions [11]. Patients with low health literacy often have difficulty communicating effectively with medical professionals, especially when treatment processes or medical conditions are explained [12]. Therefore, it is important for nurses to understand the health information literacy of elderly patients and use tailored communication strategies for elderly patients with poor health information literacy [13].

To provide high-quality medical services in nursing settings, it is important to understand the patient’s situation holistically and possess effective communication skills that take into account the patient’s characteristics and circumstances. Accurate communication is the foundation for meeting the needs and improving the satisfaction of healthcare consumers [14]. Elderly patients often require direct nursing care due to chronic diseases, dementia, and other factors. They also face the risk of pressure ulcers, urinary incontinence, long-term catheterization, and falls; thus, they often require careful and continuous observation. Furthermore, as the limited health literacy of both elderly patients and their caregivers significantly impacts medical communication and satisfaction, nurses must possess specialized communication skills to address these diverse needs [15]. In particular, decisions regarding treatment for elderly patients are often made by their families rather than the patients themselves, and consequently, nurses caring for elderly patients require frequent and close communication with their families [16]. Thus, family-related issues comprise a significant proportion of the overall nursing burden [17].

Ageism, conceptualized by Butler [18] in 1964, is systematized prejudice and discrimination against people based on their age, similar to sexism or racism, which discriminate against people based on their biological sex or skin color. Iversen [19] defined ageism as negative or positive stereotypes, prejudice, or discrimination against older people due to their chronological age, and operationalized it into cognitive (stereotypes), affective (prejudice), and behavioral dimensions (discrimination). In addition, he divided ageism into individual (micro-level), social network (meso-level), and institutional or cultural levels (macro-level), indicating that social and cultural backgrounds or perceptions are major factors that can influence ageism beyond individual ageist beliefs, feelings, and behaviors. Ageism has been shown to negatively impact depression, loneliness, chronic diseases, and subjective health status in elderly people, and can increase suicidal thoughts and decrease the quality of life in old age [20,21]. Nurses are the medical professionals who most frequently come into contact with the elderly in clinical settings, and are key personnel who should take initiative in solving health-related problems by engaging with the well-being and quality of life of the growing elderly generation [22]. However, nurses who care for the elderly are highly likely to hold negative views of them due to factors such as poor health literacy in elderly patients.

Burnout in nursing is a complex occupational syndrome resulting from chronic workplace stress that has not been successfully managed. It is characterized by three dimensions: feelings of energy depletion or exhaustion, increased mental distance from one’s job or feelings of negativism and cynicism related to one’s job, and reduced professional efficacy. As this condition stems from a lack of positive professional conditions and an overwhelming presence of negative stressors, healthcare organizations must move beyond individual-level coping and address systemic environmental factors to prevent professional erosion and ensure patient safety [23,24,25]. Nurses caring for elderly patients frequently experience patient death, are exposed to dangerous situations, require frequent observation, and experience high work burden due to repeated explanations of treatments to accommodate patients’ poor health literacy, which leads to fatigue and lack of attention to fall prevention [26]. Furthermore, caring for elderly patients requires more time and constant attention than other age groups, which places a significant burden on nurses. This, in turn, can lead to physical fatigue, depression, anxiety, fear, and sadness, which can gradually lead to a loss of motivation to work, helplessness, and burnout [27,28]. Nurses experiencing burnout are at risk of compromising their health, causing dissatisfaction with their work, and lowering the quality of patient care by developing negative professional attitudes [29].

A study conducted in 2023 [30] examined the effect of clinical nurses’ ageism on their burnout. The results showed that higher levels of ageism correlated with higher levels of nurse burnout. Given the confirmed effect of ageism on burnout, this study sought to identify which characteristics of elderly patients contribute to nurses’ ageism.

While previous studies [7,12,17,24,26,30] have examined health literacy, communication, ageism, and burnout separately, limited research has explored the interrelationships among nurses’ assessments of health information literacy in older adults, communication ability, ageism, and burnout within a single integrated framework. This study contributes to the literature by simultaneously examining these variables and identifying potential predictors of nurse burnout, thereby providing empirical evidence to inform tailored communication strategies and interventions aimed at reducing ageism and burnout in nursing practice. Building on prior research, this study aimed to examine the correlation between nurses’ assessments of health literacy in older adults, communication skills, and ageism, as well as whether these factors could be key predictors of nurse burnout. The specific objectives are as follows:

(1) To identify the general characteristics of elderly patients and the levels of health information literacy as evaluated by nurses, as well as communication ability and ageism.
(2) To identify the factors influencing health information literacy of elderly patients as evaluated by nurses, including communication ability, ageism, and burnout.
(3) To examine the correlations among health information literacy of elderly patients as evaluated by nurses, communication ability, ageism, and burnout.

## 2. Materials and Methods

To ensure ethical consideration of the participants before conducting the study, approval from the Institutional Review Board (IRB) of the institutions to which the study participants belonged was received after review, and the study was performed after explaining the purpose of the study and asking for cooperation from the hospital’s nursing headquarters. The researchers provided a written document explaining the purpose of the study, voluntary participation, and anonymity, as well as a consent form for nurses who wished to voluntarily participate in the study via a recruitment notice. Those who voluntarily consented to participate in the study signed the consent form and were requested to complete the survey.

After obtaining approval from the SJ IRB No. 1040782-250512-HR-21-157, structured self-administered questionnaires were used to collect data between 25 August 2025 and 15 September 2025. The researchers visited the hospitals and posted announcements for study participation. The questionnaires were distributed to and collected from the nurses who agreed to participate. The participating nurses were briefed about the study objectives, confidentiality of data, anonymity, and freedom to refuse or cease participation at will. Nurses spent approximately 20 min listening to the study objectives and completing the questionnaire; each nurse only completed the questionnaire once. Participating nurses were provided coupons for drinks.

The collected data were analyzed using IBM SPSS Statistics version 26.0. General characteristics of the participants and characteristics related to geriatric nursing were analyzed using descriptive statistics, including frequencies, percentages, means, and standard deviations. Differences in nurses’ assessments of older adults’ health information literacy, communication ability, ageism, and burnout according to participants’ characteristics were examined after assessing normality by calculating kurtosis and skewness and performing the Shapiro–Wilk test. Variables that did not meet the assumption of normality were analyzed using the Mann–Whitney U test and the Kruskal–Wallis test, whereas variables that satisfied normality were analyzed using the independent *t*-test and one-way analysis of variance (ANOVA). Post hoc analyses were conducted using the Scheffé or Duncan test. Levels of nurses’ assessments of older adults’ health information literacy, participants’ communication ability, ageism, and burnout were presented using descriptive statistics, including means, standard deviations, ranges, minimum values, and maximum values. Correlations among nurses’ assessments of older adults’ health information literacy, communication ability, ageism, and burnout were analyzed using Pearson’s correlation coefficient. Finally, factors influencing burnout among participants were examined using multiple linear regression analysis with the stepwise enter method.

### 2.1. Study Design

We utilized a cross-sectional study correlational design to determine the relationships among nurses’ assessments of health information literacy in older adults, communication ability, ageism, and burnout, as well as to identify factors associated with nurse burnout.

### 2.2. Participants

The participants of this study were recruited using convenience sampling from tertiary general hospitals. Eligible participants were nurses who had worked for more than 6 months in wards other than pediatric departments, operating rooms, and neonatal wards at tertiary general hospitals, had experience caring for elderly patients aged 65 years or older, and signed a written informed consent form after understanding the necessity and purpose of the study. To calculate the sample size required for regression analysis, we referred to related studies [22] and set the significance level at 0.05, effect size at 0.15, power at 0.95, and 16 predictor variables, resulting in a minimum sample size of 204. Considering a dropout rate of approximately 30%, a total of 270 questionnaires were distributed in this study, and a total of 269 questions.

### 2.3. Research Tools

#### 2.3.1. General Participant Characteristics

The general characteristics of the study participants include gender, age, education level, clinical experience, department, marital status, and place of birth. The participants’ experiences with geriatric nursing will be assessed by examining their experiences with geriatric nursing education, past experience living with an elderly person, current living with an elderly person, experience volunteering with an elderly person, anxiety about aging, and preferences for geriatric nursing.

#### 2.3.2. Assessment of Health Literacy in the Elderly

Currently, there is no established tool for measuring health literacy in Korea, and various tools have been used sporadically depending on the research. Meanwhile, due to time constraints in busy clinical settings and the potential for patients to become overwhelmed during the assessment process [31,32], formal health literacy assessment tools are not widely used. To address these issues, some studies [32,33] have investigated the effectiveness of single-item questionnaires in assessing patients’ health literacy. These studies have also reported that single-item assessments are more accurate than informal measures such as general characteristics in identifying patients with low health literacy [32]. Therefore, this study will use the following single-item questionnaire to assess the health literacy of elderly patients by nurses, referring to a previous study [32]. Specifically, we will use the following single-item questionnaire: “To what extent do you think the elderly patients you are responsible for understand nursing instructions, explanations, and health information?” The score ranges from 1 (does not understand at all) to 5 (understands completely), with a higher score indicating a higher level of medical information literacy.

#### 2.3.3. Communication Ability

Communication ability refers to how effectively one interacts with others through communication. To measure communication ability, we will use the Global Interpersonal Communication Competence (GICC) developed by Hur [34]. This research instrument consists of 15 items, and each item is answered on a 5-point Likert scale from 1 (not at all) to 5 (very much), with a higher score (1–5) indicating a higher level of communication ability. In the study by Hur [34], Cronbach’s α was 0.72, and in this study, it was 0.89.

#### 2.3.4. Ageism

Ageism refers to stereotypes (cognitive dimension), prejudices (emotional dimension), and discrimination (behavioral dimension) against older people based on chronological age, and it includes traditional psychosocial components that operate consciously or unconsciously [19]. To measure the degree of ageism, we will use the Fraboni Scale of Ageism (FSA), developed by Fraboni et al. [35], translated into Korean by Kim et al. [36], and back-translated, with the author’s permission. Fraboni et al. [35]’s FSA scale consisted of 29 items divided into three subscales: antilocution, avoidance, and discrimination [35]. However, the Korean version of the FSA scale was modified to include a total of 18 items: 7 items on emotional avoidance, 5 items on discrimination, and 6 items on stereotypes [36]. The evaluation was measured on a 4-point Likert scale, ranging from 1 point (strongly disagree) to 4 points (strongly agree). A higher total score indicates more severe ageism. At the time of development, the Cronbach’s α of the FSA scale [35] and the Korean version of the FSA scale [36] were 0.86 and 0.82, respectively, and in this study, it was 0.86.

#### 2.3.5. Burnout

We used a tool adapted by Choi Hye-yoon [37] based on Maslach & Jackson’s [38] MBI (Maslach Burnout Inventory). The MBI consists of three sub-factors: emotional exhaustion, depersonalization, and decreased self-accomplishment. It consists of 22 items, including nine items on emotional exhaustion, eight items on decreased self-accomplishment, and five items on depersonalization. The Likert-type scale ranges from 0 (never) to 6 (every day), with higher scores indicating a greater degree of burnout. Positive items were reverse-transformed. The internal consistency reliability of the instrument, Cronbach’s Alpha, was 0.76 at the time of instrument development and 0.87 in this study.

## 3. Results

### 3.1. General and Geriatric-Related Subject Characteristics

Table 1 shows the general and geriatric-related characteristics of the participants. Regarding the former, 238 (88.5%) participants were female, and most were under 29 years of age, at 154 (57.2%). In total, 232 (86.2%) had graduated from college, 189 (70.3%) were single, and 182 (67.7%) responded that they had no religion. Regarding length of employment, 133 (49.4%) had worked for 5 years or more, 67 (25.3%) had worked for 1 to 3 years, 49 (18.2%) had worked for 3 to 5 years, and 19 (7.1%) had worked for less than 1 year. A total of 126 (46.8%) participants were currently working in the internal medicine ward, and 102 (37.9%) were working in the surgical ward. Regarding geriatric-related characteristics, 231 participants (85.9%) grew up in cities, while 38 participants (14.4%) grew up in rural areas. Most of the participants responded that they had received education related to the elderly (200 participants (74.3%)), 152 participants (56.5%) had experience living with the elderly in the past, and 13 participants (4.8%) currently live with the elderly. In total, 215 participants (79.9%) had experience volunteering with the elderly, 145 participants (53.9%) were anxious about aging, 72 participants (27.1%) responded that they ‘prefer’ nursing care for the elderly, 154 participants (57.2%) responded that they felt ‘average’ in this regard, and 42 participants (15.6%) said they ‘do not prefer’ caring for elderly patients.

### 3.2. Level of Perceived Health Literacy in Elderly Patients, Communication Skills, Ageism, and Burnout Among Participants

The levels of health literacy in elderly patients evaluated by the participants, communication skills, ageism, and burnout are shown in Table 2. On a five-point scale, the mean value of the elderly patients’ health literacy was 3.46 ± 0.72, and the mean value of communication skills was 3.77 ± 0.45. The mean value of the participants’ ageism measured on a four-point scale was 2.16 ± 0.35, and the mean value of burnout measured on a six-point scale was 2.60 ± 0.80. The sub-domain scores of ageism were emotional avoidance (2.25 ± 0.47), discrimination (1.85 ± 0.40), and stereotype (2.29 ± 0.44), with stereotype being the highest. The scores for each sub-domain of burnout were 3.39 ± 1.15 points for emotional exhaustion, 1.84 ± 0.88 points for decreased self-accomplishment, and 2.37 ± 1.27 points for depersonalization.

### 3.3. Differences in Perceived Health Literacy in Elderly Patients, Communication Skills, Ageism, and Burnout Based on General and Geriatric Characteristics

Table 3 shows the differences in the health literacy of elderly patients and communication skills, ageism, and burnout in the nurses according to general and geriatric characteristics. The nurses’ assessment of health literacy showed no significant differences in the general and geriatric characteristics. Communication skills were lower in those aged 30–49 years compared to those aged 29 years or younger (F = 4.371, *p* = 0.014). Participants who reported having received education related to the elderly had higher scores than those who did not (t = 2.443, *p* = 0.015), and those who currently lived with an older adult had higher scores than those who did not (t = 2.301, *p* = 0.022). Participants who had experience volunteering with the elderly also had higher scores than those who did not (t = 1.969, *p* = 0.050). Participants who preferred geriatric nursing more often responded “preferred” than “average” or “not preferred” (F = 5.650, *p* = 0.004). Regarding ageism, the 30–49 age group showed a higher value than the 29 or younger age group (F = 4.173, *p* = 0.016), and the married group showed a higher value than the single group (t = −2.669, *p* = 0.008). The duration of employment in medical institutions was lower in the groups with less than one year of experience than in the groups with one year or more of experience (F = 8.464, *p* < 0.001). Regarding geriatric-related characteristics, the group that did not receive elderly-related education had a higher score than the group that did (t = −2.144, *p* = 0.033), the group that did not have experience volunteering with the elderly had a higher score than the group that did (t = −2.696, *p* = 0.007), the group that responded that they had anxiety about aging had a higher score than the group that responded that they did not (t = 0.505, *p* = 0.044), and the group that responded that they “did not prefer” elderly care had a higher score than the group that responded that they “preferred” or felt “average” (F = 32.33, *p* < 0.001). The difference in burnout was higher in women than in men (t = −2.803, *p* = 0.008), and the group that responded that they had no experience in volunteering with the elderly had a higher score than the group that responded that they did (t = −2.345, *p* = 0.02). The group that responded that they had anxiety about aging had a higher score than the group that responded that they did not (t = 4.110, *p* < 0.001), and the group that responded that they ‘did not prefer’ or felt ‘average’ about their preference for elderly nursing had a higher score than the group that ‘preferred’ it (F = 6.228, *p* = 0.002).

### 3.4. Correlation Coefficients Between Health Information Literacy in Elderly Patients, and Communication Skills, Ageism, and Burnout

The health literacy of the elderly showed a positive correlation with the communication skills of the participants (r = 0.13 (0.04)) and a negative correlation with ageism and burnout (r = −0.144 (0.02) and r = −0.140 (0.02), respectively). Communication skills showed a negative correlation with both ageism and burnout (r = −0.30 (*p* < 0.001) and r = −0.37 (*p* < 0.001), respectively), and there was a positive correlation between ageism and burnout (r = 0.43 (*p* < 0.001) (Table 4).

### 3.5. Factors Influencing Burnout in Participants

Before performing the regression analysis, the autocorrelation of the dependent variable and the multicollinearity among the independent variables were examined. The Durbin–Watson index was used for autocorrelation, the value of which was 2.015, which is between the standard values of 1 and 3, confirming independence without autocorrelation. The multicollinearity among the independent variables was examined using the tolerance limit and variance inflation factor (VIF). The tolerance limit was 0.770~0.975, which is more than 0.1, and the variance inflation factor was 1.043~1.289, which is less than 10, so there were no multicollinearity problems. Therefore, these data were suitable for conducting a regression analysis, and as a result of using the all-selection method, communication ability (B = −0.458, *p* < 0.001), ageism (B = 0.650, *p* < 0.001), gender (female) (B = 0.345, *p* = 0.010), and aging anxiety (B = −0.295, *p* = 0.001) showed that lower communication ability, higher ageism, being female, and having aging anxiety (Yes = 1) were associated with higher burnout. The explanatory power of these variables to explain burnout was 29.3% (Adjusted R^2^ = 0.293). Placing greater interpretative emphasis on predictors with higher standardized β values, ageism (β = 0.287) shows the strongest association with burnout, followed by communication ability (β = −0.251), gender (β = 0.139), and aging anxiety (β = −0.181) (B-value: communication −0.458, ageism 0.650, gender 0.345, aging anxiety −0.292) (Table 5).

## 4. Discussion

This study was conducted to investigate the relationships among perceived health literacy in elderly patients, communication skills, ageism, and burnout among ward nurses, and to identify factors influencing burnout. The results showed that nurses’ perceptions of health literacy in elderly patients, communication skills, and ageism were significantly correlated with burnout. The main predictors of burnout were communication skills, ageism, gender, and anxiety about aging.

In this study, the average health literacy score of older adults, as assessed by nurses, was 3.46 ± 0.72 out of 5. The Single Item Literacy Screener (SILS), used as an assessment tool, has been reported to be effective for patients requiring reading assistance in clinical settings, but no direct evaluation results have been reported for older adults to date. Therefore, direct comparisons are difficult. However, in a study using the Single Item Literacy Screener (SILS), the average health literacy score of elderly patients was between 3 and 5, indicating a significant need for assistance in reading and understanding health information. Given these diverse needs and the impact of limited literacy on medical communication, nurses must possess more specialized communication skills to ensure effective patient care [39]. Furthermore, in this study, lower health literacy scores were associated with higher levels of ageism and burnout. This suggests that low health literacy in older adults is associated with a nursing environment characterized by the need for additional explanations, repetitive communication, and frequent monitoring during tasks. Previous studies have also reported that poor health literacy leads to ineffective communication with medical staff and negatively affects health outcomes [5,8], and that nurses experience high stress while caring for these patients [26], which is consistent with the results of this study.

The nurses’ communication skills averaged at 3.77 ± 0.45 points, which was slightly higher than the 3.30 ± 0.43 points [40] and 3.33 points [41] reported in other studies using the same instrument. However, this result is similar to those of most previous studies that found moderate or higher levels of communication skills. This is thought to be due to the nature of nurses’ work, which requires them to communicate and collaborate with various medical professionals in clinical settings while providing explanations and education to patients and their guardians. In relation to the general and geriatric-related characteristics of the participants, communication skills were significantly higher in the younger age groups, those who had received geriatric-related education, those who currently lived with elderly people, those who had experience volunteering with the elderly, and those who preferred geriatric care. The higher communication skills in the younger group may be due to the fact that these nurses had received interpersonal relationship and communication education in their university curriculum, whereas nurses aged 30 years or older reported that they rarely utilized formal resources such as communication programs or self-development training after graduation. In addition, as frequent interaction with the elderly through cohabitation or volunteering enhances understanding and communication skills, nurses must cultivate specialized competencies to overcome health literacy barriers and address the diverse needs of elderly patients and their families [42,43]. Poorer communication skills were associated with higher burnout. Communication between healthcare providers and patients is a key element of patient satisfaction and treatment experience [14], and more specialized and detailed communication skills are essential, especially for elderly patients with chronic diseases and cognitive decline. This suggests that communication skills are essential for nurses to provide high-quality care by continuously paying attention to the responses of not only elderly patients but also their caregivers and maintaining therapeutic relationships. The finding that higher levels of burnout were observed among nurses with lower communication skills indicates an association between communication skills and burnout in the context of coordinating the diverse needs of patients and their caregivers and providing care.

The mean ageism score of this study’s participants was 2.16 ± 0.35 out of 4, which was similar to the score of 2.21 in a study conducted on nurses in general hospitals [21] and the 2.06 in a study conducted on nurses in domestic tertiary hospitals [44], and lower than the 2.63 points reported in another study on general hospital nurses. However, the moderate elderly ageism score is considered in the same context as a previous study [14], which interpreted that nurses perceive the elderly as objects of care as professional healthcare personnel and maintain a relatively neutral attitude toward them. Analyzing the degree of ageism according to the general and geriatric-related characteristics of the participants showed that the ageism score was higher in nurses aged 30–49 years than in those aged 29 years or younger. This result was similar to that of a study that reported that the ageism score of nurses in their 40s was statistically significantly higher than that of nurses in their 20s and that it increased with age [45]. Married individuals had higher ageism scores, which differs from a study [46] that found no significant difference in ageism by marital status. This suggests the need for education to enhance nurses’ awareness of older people’s needs. The higher ageism scores in those with more than three years of experience are thought to be due to their increased contact with older adults, which can bias their experiences with older adults with complex chronic diseases [22]. Higher ageism scores were found in those who had not received education related to the elderly, had no experience volunteering with the elderly, had anxiety about aging, and did not prefer elderly care. This reflects the findings that those with more contact or more positive experiences with the elderly are less likely to have an ageist perspective [3], and is similar to findings [3] that more negative attitudes toward the elderly are positively correlated with higher ageism scores. Therefore, it is necessary to investigate nurses’ perceptions of older adults in clinical settings and to strive to reduce the level of ageism in clinical practice. Ageism was found to be the strongest predictor of burnout (β = 0.287), suggesting that nurses’ stereotypes, prejudices, and discriminatory feelings toward older adults can exacerbate emotional exhaustion and reinforce negative attitudes during caregiving, creating a vicious cycle. Previous studies have also shown that negative perceptions of older adults increase nurses’ burden of work and stress [20,21] and are directly linked to burnout, particularly in geriatric care situations where repetitive explanations, uncertainty about prognosis, and conflict with patients and their families are frequent [27,28]. According to a recently reported scoping review on ageism toward older adults in the healthcare field, neoliberal logic that devalues older adults perpetuates the spread of negative images and attitudes toward them. These perceptions are transferred into healthcare professionals’ daily service practices through the formation of stereotypes (thoughts), prejudices (feelings), and discrimination (actions). Furthermore, such ageism manifests at both interpersonal and institutional levels and permeates healthcare professionals’ work processes [46]. Therefore, it is necessary to develop and implement various educational programs that promote correct understanding of older patients and address misconceptions about them to reduce ageism.

It is also significant that nurses with anxiety about aging experienced higher levels of burnout. Anxiety about aging can increase negative emotions and cognitive biases when dealing with older patients, potentially leading to emotional exhaustion. Nurses with experience in geriatric education and volunteer work showed higher communication skills and lower levels of ageism and burnout. This suggests that repeated experience working with older adults can foster positive perceptions of older adults and contribute to practical competency enhancement. This highlights and supports the need to develop an e-learning module for improving expertise in geriatric nursing.

In analyzing the factors influencing nurse burnout, a regression model that included factors such as low communication skills, high ageism, female gender, and anxiety about aging was significant, explaining approximately 29% of the variance. Therefore, improving communication skills and ageism, key elements of geriatric nursing, were identified as a key strategy for preventing nurse burnout and improving the quality of nursing. This highlights the importance of practical, practice-oriented education applicable to actual clinical situations, beyond simple attitude correction or knowledge transfer.

While this study produced meaningful results, it has the following limitations, which should be addressed in future research. First, as this study was conducted using a cross-sectional design, there is a risk of causal inference bias; therefore, longitudinal studies or repeated-measures designs are needed to better track the impact of changes in ageism on burnout. Furthermore, the study participants were limited to nurses at a single hospital, limiting the generalizability of the findings to the entire nursing population. Finally, because this study assessed only nurses’ perceptions of older adults’ health literacy, there may be discrepancies with the actual characteristics of older adults. In addition, the single-item questionnaire, a tool used by nurses to obtain health information from elderly participants, is an abbreviated version of the Short Test of Functional Health Literacy Assessment (S-TOFHLA). It is a useful tool for determining the extent to which participants require assistance to understand complex health information, but it has limitations in that the use of this measure may vary depending on the extent of need required by the subject.

## 5. Conclusions

This study examined the relationships between health literacy in elderly patients, communication skills, agism, and burnout among ward nurses. It also analyzed the factors influencing burnout. The results identified low communication skills, high agism, being female, and anxiety about aging as major factors contributing to burnout, with agism being the most influential factor.

These results suggest that nurses’ professional communication skills and positive perceptions of older adults can mitigate burnout and improve the quality of care. Furthermore, by confirming that education and experience related to the elderly are effective in improving these factors, the study suggests the need for the development of systematic and standardized training programs to enhance professionalism in geriatric nursing.

## Figures and Tables

**Table 1:** General and geriatric-related subject characteristics.

Variables	Categories	n (%) or M ± SD
Gender	Male	31 (11.5)
	Female	238 (88.5)
Age (yr)		30.80 ± 7.385
	≤29	154 (57.2)
	30~49	101 (37.5)
	≥50	14 (5.2)
Level of education	Diploma	20 (7.4)
	Bachelor’s degree	232 (86.2)
	Above Master’s degree	17 (6.3)
Marital status	Single	189 (70.3)
	Married	80 (29.7)
Religion	Yes	87 (32.3)
	No	182 (67.7)
Work experience (yr)		84.68 ± 92.513
	0.6~<1	19 (7.1)
	≤1~<3	67 (25.3)
	≤3~<5	49 (18.2)
	≤5	133 (49.4)
Current department	Internal ward	126 (46.8)
	Surgical ward	102 (37.9)
	ICU	2 (0.7)
	ER	1 (0.4)
	Others	38 (14.1)
Area of residency while growing up	Urban	231 (85.9)
	Rural	38 (14.1)
Geriatric education	Yes	200 (74.3)
	No	68 (25.3)
Residential experience with elders	Yes	152 (56.5)
	No	117 (43.5)
Residential status with elders	Yes	13 (4.8)
	No	256 (95.2)
Volunteer experience with elders	Yes	215 (79.9)
	No	54 (20.1)
Aging anxiety	Yes	145 (53.9)
	No	124 (46.1)
Preference for geriatric nursing	Preferred	73 (27.1)
	Average	154 (57.2)
	Not preferred	42 (15.6)

**Table 2:** The level of perceived health literacy in elderly patients, communication skills, ageism, and burnout of participants.

Variables	Range	M ± SD	Minimum	Maximum	Skew	Kurtosis
Health literacy in elderly patients		3.46 ± 0.72	1	5	−0.952	−0.126
Communication skills		3.77 ± 0.45	2.47	5.00	−0.222	0.289
Ageism	0–4	2.16 ± 0.35	1.22	3.22	−0.057	0.369
Emotional avoidance	0–4	2.25 ± 0.47	1.00	3.86	−0.105	0.499
Discrimination	0–4	1.85 ± 0.40	1.00	2.80	0.008	−0.671
Stereotyping	0–4	2.29 ± 0.44	1.00	4.00	0.066	0.868
Burnout	0–6	2.60 ± 0.80	0.18	4.41	−0.402	−0.240
Emotional exhaustion	0–6	3.39 ± 1.15	0.00	5.78	−0.503	−0.147
Personal accomplishment	1–6	1.84 ± 0.88	0.00	4.75	0.397	−0.175
Depersonalization	1–6	2.37 ± 1.27	0.00	5.60	−0.28	−0.815

**Table 3:** Differences in perceived health literacy in elderly patients, communication skills, ageism, and burnout based on general and geriatric characteristics.

Variables	Categories	Health Literacy in Elderly Patients		Communication Skills		Ageism		Burnout	
		M ± SD	t/F(*p*)	M ± SD	t/F(*p*)	M ± SD	t/F(*p*)	M ± SD	t/F(*p*)
Gender	Male	3.52 ± 0.72	0.46 (0.65)	3.85 ± 0.41	1.05 (0.30)	2.08 ± 0.45	−1.25 (0.21)	2.15 ± 0.96	−2.80 (0.008)
	Female	3.45 ± 0.72		3.76 ± 0.45		2.17 ± 0.34		2.65 ± 0.76	
Age (yr)	≤29 ^a^	3.55 ± 0.68	1.51 (0.22)	3.83 ± 0.46	4.37 (0.01) a > b	2.10 ± 0.35	4.17 (0.02) a < b	2.65 ± 0.82	1.41 (0.25)
	30~49 ^b^	3.37 ± 0.74		3.67 ± 0.40		2.23 ± 0.35		2.56 ± 0.80	
	≥50	3.64 ± 0.75		3.90 ± 0.53		2.24 ± 0.31		2.29 ± 0.58	
Level of education	Diploma	3.65 ± 0.67	1.10 (0.33)	3.83 ± 0.59	0.93 (0.40)	2.13 ± 0.33	0.12 (0.88)	2.49 ± 0.81	1.09 (0.34)
	Bachelor’s degree	3.43 ± 0.73		3.76 ± 0.43		2.16 ± 0.36		2.62 ± 0.81	
	Above Master’s degree	3.59 ± 0.71		3.89 ± 0.47		2.18 ± 0.37		2.35 ± 0.57	
Marital status	Single	3.49 ± 0.69	0.85 (0.40)	3.79 ± 0.45	0.99 (0.32)	2.12 ± 0.35	−2.67 (0.008)	2.61 ± 0.83	0.53 (0.60)
	Married	3.44 ± 0.79		3.73 ± 0.44		2.24 ± 0.34		2.55 ± 0.73	
Religion	Yes	3.51 ± 0.72	0.80 (0.43)	3.81 ± 0.46	0.94 (0.35)	2.15 ± 0.33	−0.26 (0.79)	2.59 ± 0.81	−0.04 (0.97)
	No	3.44 ± 0.73		3.75 ± 0.44		2.16 ± 0.36		2.60 ± 0.80	
Work experience (yr)	0.6~<1 ^a^	3.63 ± 0.50	1.48 (0.22)	3.94 ± 0.56	1.24 (0.30)	1.81 ± 0.35	8.46 (0.000) a < b, c, d	2.28 ± 0.90	1.14 (0.33)
	1≤~<3 ^b^	3.48 ± 0.69		3.80 ± 0.42		2.11 ± 0.33		2.66 ± 0.91	
	≤3~<5 ^c^	3.59 ± 0.64		3.74 ± 0.49		2.22 ± 0.33		2.63 ± 0.72	
	≤5 ^d^	3.38 ± 0.79		3.74 ± 0.42		2.21 ± 0.34		2.59 ± 0.75	
Current department	Internal ward ^a^	3.53 ± 0.70	1.03 (0.36)	3.79 ± 0.41	1.32 (0.27)	2.16 ± 0.34	0.36 (0.70)	2.56 ± 0.83	0.77 (0.47)
	Surgical ward ^b^	3.40 ± 0.72		3.79 ± 0.47		2.17 ± 0.39		2.59 ± 0.78	
	ICU and others	3.41 ± 0.77		3.67 ± 0.48		2.12 ± 0.29		2.74 ± 0.77	
Area of residency while growing up	Urban	3.46 ± 0.72	−0.02 (0.99)	3.66 ± 0.60	−0.95 (0.35)	2.10 ± 0.20	−0.81 (0.42)	2.50 ± 0.94	−0.62 (0.54)
	Rural	3.46 ± 0.73		3.95 ± 1.55		2.16 ± 0.36		2.60 ± 0.79	
Geriatric education	Yes	3.47 ± 0.74	0.53 (0.59)	3.81 ± 0.43	2.44 (0.02)	2.13 ± 0.36	−2.14 (0.33)	2.52 ± 0.81	−2.62 (0.009)
	No	3.42 ± 0.68		3.65 ± 0.47		2.24 ± 0.32		2.81 ± 0.75	
Residential experience with elders	Yes	3.45 ± 0.76	−0.27 (0.79)	3.77 ± 0.48	−0.01 (0.99)	2.14 ± 0.38	−1.02 (0.31)	2.60 ± 0.81	0.01 (0.99)
	No	3.47 ± 0.67		3.77 ± 0.40		2.18 ± 0.32		2.59 ± 0.80	
Residential status with elders	Yes	3.62 ± 0.51	0.79 (0.43)	4.05 ± 0.46	2.30 (0.02)	1.99 ± 0.32	−1.74 (0.08)	2.39 ± 0.53	−0.94 (0.35)
	No	3.45 ± 0.73		3.76 ± 0.44		2.17 ± 0.35		2.61 ± 0.81	
Volunteer experience with elders	Yes	3.48 ± 0.71	0.73 (0.47)	3.80 ± 0.43	1.97 (0.05)	2.13 ± 0.35	−2.70 (0.007)	2.54 ± 0.81	−2.35 (0.02)
	No	3.40 ± 0.77		3.66 ± 0.49		2.27 ± 0.34		2.83 ± 0.71	
Aging anxiety	Yes	3.42 ± 0.75	−1.08 (0.28)	3.76 ± 0.44	−0.33 (0.75)	2.20 ± 0.34	0.51 (0.04)	2.78 ± 0.68	4.11 (0.000)
	No	3.51 ± 0.68		3.78 ± 0.45		2.11 ± 0.36		2.38 ± 0.88	
Preference for geriatric nursing	Preferred ^a^	3.52 ± 0.74	0.36 (0.70)	3.92 ± 0.48	5.65 (0.004) a > b, c	1.94 ± 0.37	32.33 (<0.001) a, b < c	2.34 ± 0.85	6.23 (0.002) a < b, c
	Average ^b^	3.43 ± 0.68		3.73 ± 0.41		2.19 ± 0.27		2.65 ± 0.74	
	Not preferred ^c^	3.45 ± 0.74		3.68 ± 0.46		2.42 ± 0.37		2.85 ± 0.84	

The superscript letters(a, b, c, d) are for the following statistical analyses: When significant differences were found among variables (e.g., the demographic characteristic “Age (yr)” had a significant F (p) value of 4.37 (0.01) for Communication Skills), additional post-hoc analyses were conducted. Different letters indicate statistically significant differences.

**Table 4:** Correlation coefficients between health information literacy in elderly patients and communication skills, ageism, and burnout.

Variables	Health Literacy in Elderly Patients	Communication Skills	Ageism	Burnout
Health literacy in elderly patients	1	0.13 * (0.04)	−0.144 * (0.02)	−0.140 * (0.02)
Communication skills		1	−0.30 ** (*p* < 0.001)	−0.37 ** (*p* < 0.001)
Ageism			1	0.43 ** (*p* < 0.001)
Burnout				1

* < 0.05, ** < 0.01.

**Table 5:** Factors influencing burnout in participants.

Variables	B	95% CI	β	t	*p*	F(*p*)	*adj R* ^2^
(constant)	2.860	[1.693, 3.998]				14.391 (<0.001)	0.293
Health literacy in elderly patients	−0.047	[−0.169, 0.062]	−0.042	−0.797	0.426		
Communication skills	−0.458	[−0.654, −0.255]	−0.251	−4.520	0.000		
Ageism	0.650	[0.383, 0.911]	0.287	4.841	0.000		
Gender (F = 1, M = 0)	0.345	[0.114, 0.641]	0.139	2.603	0.010		
Geriatric education (Yes = 1, No = 0)	0.120	[−0.096, 0.301]	0.065	1.191	0.235		
Experience volunteering with elders (Yes = 1, No = 0)	0.056	[−0.153, 0.286]	0.028	0.502	0.616		
Aging anxiety (Yes = 1, No = 0)	−0.292	[−0.477, −0.132]	−0.181	−3.343	0.001		
Preference for geriatric nursing (No = 1, Yes= 0)	0.103	[−0.125, 0.360]	0.047	0.833	0.405		

## Data Availability

Data is contained within the Supplementary Materials.

## Supplementary Materials

The following supporting information can be downloaded at: https://www.mdpi.com/article/10.3390/nursrep16020045/s1, the original questionnaire (English version), dataset of questionnaire.

## Public Involvement Statement

There was no public involvement in any aspect of this research.

## Guidelines and Standards Statement

This manuscript was drafted against the STROBE checklist for cross-sectional research.
