# Supplementary material for: The Effect of Elderly Patients’ Health Information Literacy, Ageism, and Communication Skills on Clinical Nurses’ Burnout: A Cross-Sectional Study
Source: Nurs Rep. 2026 Jan 29;16(2):45. doi: 10.3390/nursrep16020045 (PMC12943585; doi:10.3390/nursrep16020045)
Supplement: Supplementary file 1 [file nursrep-16-00045-s001.zip › The original questionnaire (English version).pdf]

Version 1.0, 05/10/2025

All information collected in this survey will be used for purposes other than statistical purposes and will remain confidential, as stipulated in the Statistics Act (Articles 33 and 34).

ID			

The effect of elderly patients' health information literacy, ageism, and communication skills on clinical nurses' burnout

Hello,

This survey aims to investigate the impact of geriatric patients' health literacy, ageism, and communication skills on burnout among clinical nurses.
Your honest responses will provide valuable data on the impact of geriatric patients' health literacy, ageism, and communication skills on burnout among clinical nurses. They will also serve as a valuable foundation for developing a system to predict burnout among ward nurses in clinical settings. They will not be used for any other purpose.
You have the right to refuse to participate in this survey, and there will be no disadvantages to not participating. You may also withdraw your participation at any time during the study.

Your responses will be used solely for research purposes, and your personal information will be strictly protected in accordance with Article 33 of the Statistics Act. Therefore, to ensure accurate data collection, please answer all questions honestly and completely.
Thank you for your participation.

If you have any questions while completing the survey, please contact the principal investigator listed below.

May 2025

▪ Principal Investigator: Prof. Eunhee Shin, Department of Nursing, Sangji University
(Contact: 010-6278-9622)

Research Consent Form

I fully understand the purpose of this study and consent to the use of my responses. I also confirm that I have been informed that I may withdraw my consent at any time.

Agree ☐ Disagree ☐

Name: (Signature)

※ The following questions are about communication. Please mark (√) the appropriate answer based on your usual thinking.

	Strongly disagree	Disagree	Neutral	Agree	Strongly agree
1. I let my friends know who I really am.					
2. I can see things from other people's perspectives.					
3. I feel comfortable in many situations.					
4. I assert my rights and opinions.					
5. When I talk, I listen carefully to what the other person is saying.					
6. I lead the conversation by discussing what topics to cover.					
7. I express myself well, both verbally and nonverbally.					
8. People say I'm a warm person.					
9. My friends believe that I care about them.					
10. I am effective at conversation.					
11. I use polite and informal speech appropriately depending on the person I am speaking to.					
12. I speak logically.					
13. I can easily figure out what the other person is trying to say during a conversation.					
14. When I'm talking, I let the other person know that I understand what they're saying by speaking or nodding.					
15. I create an environment where I can focus on the conversation.					

※ The next question is about **ageism.** Please put a √ in the box that applies to your opinion.

	Strongly disagree	Disagree	Agree	Strongly agree
1. I personally would not want to spend much time with an old person.				
2. I would prefer not to go to an old people’s gathering, if invited.				
3. I would prefer not to live with an old person.				
4. It's a lot of fun to be with an old person.				
5. I don't like it when old people try to make conversation with me.				
6. Most old people are interesting and individualistic.				
7. I often avoid eye contact with old people.				
8. It is best for older people to live apart so as not to bother others.				
9. Most old people should not renew their driver's licenses.				
10. Seniors have virtually no need for local sports facilities.				
11. Older people deserve the same freedoms and rights as other members of our society.				
12. It's sad to hear about the plight of old people.				
13. Most older people live trapped in the past.				
14. When you're around older people, it's probably normal to feel depressed.				
15. Old people complain more than young people.				
16. Most older people are not interested in making new friends, but rather prefer to spend time with friends they have known for a long time.				
17. Most older people are happiest when they are with people of similar age.				
18. Most older people try to save up their money or possessions rather than giving them away.				

※ The next question is about **psychological burnout**. Please mark (√) the appropriate box according to your usual thinking.

	None at all	Less than once a year	Less than once a month	a few times a month	Once a week	a few times a week	every day
1. I feel mentally exhausted from work.							
2. I'm exhausted at the end of a long day's work.							
3. I wake up in the morning and feel discouraged when I think about having to go to work again today.							
4. I can easily understand the subject's feelings.							
5. I feel like I'm treating some of my subjects like objects without personalities.							
6. Working with people all day is really hard for me.							
7. I deal effectively with the subject's problems.							
8. I am completely exhausted from my work.							
9. I feel like I am making a positive impact on other people's lives through my work.							
10. I've become more and more insensitive to people since I've been in this job.							
11. I'm afraid this will make me emotionally dry.							
12. I feel very energetic.							
13. I feel frustrated with my work.							
14. I feel like I'm working too hard.							
15. I don't really care what happens to any subject.							
16. It is very stressful for me to work while dealing with people directly.							
17. I can easily create a comfortable atmosphere for the subject.							
18. I feel very fulfilled after working closely with the subject.							
19. I have accomplished many valuable things through this work.							
20. There are times when I feel like I'm helpless.							
21. I deal with emotional problems very calmly while working.							
22. I feel that the subjects are blaming me for some of their problems.							

※ The following questions are about health literacy in older adults. Please mark (√) the appropriate box according to your usual perception.

	Does not understand at all	Does not understand	Understands somewhat	Understands partially	Understands completely
To what extent do you think the elderly patients under your care understand the nurse's instructions, explanations, and health information?					

※ Here are some questions about your general characteristics. Please fill in each question.

1. Gender?
   ____① Male ____② Female

2. Age? ____________year

3. Which of the following is your educational background?
   ____① College ____② University ____③ Master's degree or higher

4. What is your marital status?
   ____① Single ____② Married

5. Do you have a religion?
   ____① Yes ____② No

6. What is your working period at the current workplace? ____________month

7. Which type of healthcare institution do you currently work at?
   ____① Tertiary hospital ____② General or specialized hospital
   ____③ Long-term care hospital ____④ Other ________________

8. Which department are you currently working in?
   ____① Internal ward ____② Surgical ward
   ____③ Intensive care unit(Internal/Surgical) ____④ Outpatient
   ____⑤ Emergency room ____⑥ Others ________________

9. What type of area did you live in while growing up?

____① metropolitan areas ____② Local city

____③ Rural area ____④ Others ( )

10. Have you received any educational related to old patients?

____① Yes ____② No

11. Have you ever lived with an old person in the past?

____① Yes ____② No

12. Are you currently living with an old person?

____① Yes ____② No

13. Have you ever participated in volunteer work related to old people?

____① Yes ____② No

14. Have you ever felt anxious about aging?

____① Yes ____② No

15. Which of the following are your preferences for nursing care for older patients?

____① Very preferred ____② Preferred

____③ Average ____④ Not preferred

____⑤ Not very preferred

♣ Thank you very much for your response!! ♣
